# The Regional Migration-Development Nexus in Australia: What Migration? Whose Development?

**DOI:** 10.3389/fsoc.2021.602487

**Published:** 2021-02-11

**Authors:** Martina Boese, Anthony Moran

**Affiliations:** Department of Social Inquiry, La Trobe University, Bundoora, VIC, Australia

**Keywords:** Australia, employment, migration-development nexus, migration policies, multiculturalism, refugees, rural migration, temporary migration

## Abstract

Both regional resettlement of refugees, and the attraction of different kinds of migrant labor to regional areas, have been significant trends in Australia’s recent migration policies. Using the concept of the migration-development nexus, we address important questions about the nature and scope of development these different policies aim to promote, and achieve. We examine the intersection of policies and initiatives implemented to encourage and support refugee settlement and regional migration in Australia with the perspectives of regionally settled migrants and refugees on their regional migration outcomes. We argue that recent government policies, and multi-stakeholder initiatives aimed at regional migration and/or settlement, cast migrants as differential contributors to regional development, useful either in terms of their skills (skilled migrants) or their labor (backpackers, seasonal workers, refugees). The co-presence of different groups of migrants in regional locations is also shaped by the fluctuating employer demands for mobile labor in combination with visa regulations. We draw on data from three projects on regional settlement, multiculturalism and mobilities to analyze three important elements of regional migration that are central to a critical analysis of the nexus between rural migration and development in regional Australia: the complex roles of employers; the embedding of regional migration in migrants’ life courses; and the tension between long-term migration outcomes and quick fixes. By focusing on development as it is experienced by migrants themselves and interpreted by different stakeholders in regional migration, we draw attention to the limitations of a purely instrumental view of migrants as agents of regional development. We argue that the sustainability of regional migration policies will depend on recognizing the important role of migrants’ hopes, needs and aspirations as well as their rights, and the unintended human costs and consequences of exclusively economically driven migration policy design.

## Introduction

Regional resettlement of refugees, and attracting migrant workers to regional areas, are significant recent trends in Australian migration policies, raising important questions about the nature and scope of development these different policies aim to achieve, and actually achieve. Increasingly, all levels of government, policy consultants, think tanks and scholars cast regional migration^1^ as a “win-win” scenario in Australia. Rural communities experiencing population decline and labor and skills shortages are seen as benefitting from migrant settlement and labor (Regional Australia Institute, 2018; Tudge, 2019; Joint Standing Committee on Migration, 2020). Migrants and refugees are said to find employment more easily than in urban locations (similar to other countries, traditionally the major settlement locations for new arrivals). What is less clear than governmental promotion of regional and rural migration, and the acknowledged contribution of migrants and refugees to local economies and productivity in regional Australia (Collins et al., 2016), is how migrants themselves experience the outcomes of regional migration. These experiences are, however, relevant for the social and economic sustainability of such policies and initiatives, which is evident in policy makers’ concern with migrants’ retention in regional areas (Wulff and Dharmalingam, 2008; Krivokapic-Skoko and Collins, 2016). This paper investigates the relationship between regional migration policy aims and the varied experiences of different categories of migrants at the center of the regional migration-development nexus. The key question pursued is adapted from Raghuram's (2009) question on the nexus between migration and international development^2^. Raghuram draws attention to migrants’ own development rather than the development of their countries of origin in assessing the development outcomes of international migration. We suggest in a similar manner to extend the question about the development outcomes of regional migration beyond regional economies to migrants themselves. In other words, what kind of development can regional migration afford to migrants? We explore this question by asking more specifically: how is regional migrants’ development shaped by employers? How can we assess the development outcomes of regional migration in the context of migrants’ life courses? And finally, how does the tension between short- and long-term outcomes affect an assessment of development via regional migration?

This paper provides a combined analysis of policies and initiatives devised to either encourage labor migration or increase refugee settlement in rural Australia; perspectives on rural migration outcomes of key stakeholders involved with migration or settlement support processes; and perspectives of regionally settled migrants and refugees themselves. Drawing on our own research into regional settlement in Australia conducted between 2010 and 2020, we posit that a better understanding of the development outcomes of rural and regional migration requires consideration of diverse, partly intersecting and partly opposed interests of different stakeholders and how they shape migration outcomes. Our analysis of the experience of regional migration and settlement outcomes according to migrants and refugees, accessed through their own accounts in interviews and focus groups, and according to other stakeholders in regional Australia, also highlights the importance of studying the different target groups of regional migration and settlement as co-residents and as social and economic actors with varying hopes, needs and aspirations.

We begin with an overview of regional migration and settlement policies, distinguishing between regulation of regional labor migration (“skilled” and “unskilled”), and of refugee resettlement in Australia. Next, we review key research findings on regional migration experiences for migrants and refugees in Australia in the context of international scholarship on rural migration, focusing on analysis of employment, and socio-cultural dimensions of regional settlement. We introduce the “migration-development nexus” as a lens for analyzing outcomes of regional migration. After describing the research projects on which this paper draws, we discuss three influences on regional migration outcomes, which in our view need to be considered alongside each other in an analysis of the regional migration-development nexus. Firstly, the central and multidimensional roles of employers as key stakeholders and beneficiaries of regional migration; secondly, the embedding of regional migration in migrants’ life courses; and thirdly, the tension between long-term regional migration outcomes and quick fixes. The paper concludes by highlighting the risks of reducing regional migrants to agents of regional economic development and by arguing for a combined analysis of different target groups of regional migration policies.

## Australian Regional Migration and Regional Refugee Settlement: A Policy Review

The Australian government separated its migration program from its humanitarian program in 1983 to distinguish its humanitarian obligations from its domestic, social and economic migration goals (Galligan et al., 2014). Economic imperatives have shaped Australian migration policies since the 1980s (Walsh, 2011) and regional migration policies strongly reflect this. Australia is internationally renowned as a highly selective, skills-oriented and demand-based immigration country, second after Canada to introduce a points-test to screen migrants based on their qualifications, language skills and professional experience. While family migration has persisted alongside skills-based migration, the latter has dominated the migrant intake since the late 1990s, evident in a disproportionate growth of permanent skilled, business and, more recently, temporary migration. Within the “skilled migration” category, employers are an ever-growing influence on intake through the relative and absolute growth of employer-sponsored visas and the introduction of skills-lists based on employer demand (Galligan et al., 2014). Finally, the generous admission of temporary migrants since the late 1990s also followed the rationale of admitting the migrants needed by specific Australian industry sectors as long as they are required by employers, without granting them the social entitlements enjoyed by permanent residents. At any point there is an estimated one million temporary visa holders in Australia (Mares, 2016), which also reveals another lesser known dimension of Australia’s migration policies. Alongside nominal labor migration programs such as the temporary skilled program, temporary visa holders include several other groups not screened through occupation-lists and points-tests, yet providing critical and numerically significant pools of labor for unskilled and low-skilled jobs in sectors such as hospitality, horticulture and food processing. This includes holders of international student visas, Working Holiday- and Work and Holiday-visas which are thus de facto labor migration programs, expanding the economic orientation of Australia’s migration policies beyond dedicated and skills-focused labor migration programs (Tham et al., 2016).

Regional and rural Australia is where all the mentioned categories of migrants are in high demand and where government policies have aimed to direct them. In a settler colonial country, overseas migration to regional locations goes back to Australia’s initial colonization (Butler and Ben, 2020). Migrants and migrant labor in particular have shaped regional Australia long before the more recent targeted regional migration policies, from the long presence of Chinese migrants in regional Victoria to the forced farm labor of Pacific Islanders from the 1860s in Queensland, known as “blackbirding” (Stead and Altman, 2019) to different waves of seasonal workers in the 20th century across Australia. However, from the late 1990s until now, the rural migration of “skilled migrants” has been incentivized through different regional migration visas, skilled regional visas (most recently, Australian Government, 2019), and also state-led regional skilled migration initiatives (for example State Government of Victoria, 2007). The lower points-threshold for regional visas has been an important attraction for applicants, who are variably tied to a specific–regionally based - employer or to a regional location for the visa-duration. Alongside these policies, the already mentioned Working Holiday (WHV)- and Work and Holiday-visas have provided key policy tools to attract “flexible” and highly mobile migrant labor to orchards, farms, packing sheds and hospitality businesses in regional Australia. The 12-months WHV, in particular, comes with the option to apply for a second year-visa on the condition of having worked for 88 days in a regional location in the first year. Regional employer demand for horticultural workers has also been addressed by the Seasonal Worker Program (piloted in 2009) and the Pacific Labor scheme (introduced in 2018) which brings workers from several Pacific Islands^3^ for fixed-term periods to employers who participate in the Scheme (Australian Government, 2020), intended to produce development benefits via remittances for the participant Pacific Island state economies (World Bank, 2006). It is worth noting that the earlier discussed skilled visas allow migrants to bring their partners and dependents, while the later-mentioned Working Holiday, Seasonal worker- and Pacific Labor schemes do not, expecting the workers to depart again ‐ and (in the case of the Seasonal worker and Pacific Labor schemes) to invest in ‐ their overseas home.

Alongside these policies, the federal government in Australia has also implemented the direct resettlement of “unlinked” refugees (i.e., refugees without pre-existing family connections in Australia) in selected regional towns in Victoria, New South Wales and Queensland since the early 2000s, led by the Department of Home Affairs and its predecessors. Other groups of former refugees have been relocated from metro Melbourne to selected regional towns, in joint initiatives of some city-based community sector organizations and rural employers, local governments and/or service providers (see f. ex. Taylor and Stanovic, 2005). Some of these initiatives have turned out to be short-lived while a few others have been hailed as successful blueprints for rural revitalization through refugee settlement (AMES Australia and Deloitte Access Economics, 2015). Efforts to facilitate the relocation of refugees from capital cities to regional and rural towns have grown over the last 15 years, and many metropolitan-based refugees who struggle to find work or suitable accommodation in major cities like Melbourne or Sydney have independently followed the promise of a better life in a small rural town with more accessible employment and affordable housing.

The mentioned economically-driven policies and initiatives have been paralleled by local government efforts, that are primarily concerned with the social and cultural impacts of migration to regional towns and communities (Boese and Phillips, 2017). Over the past 10 years, many regional cities and towns in Australia have developed local initiatives to support the settlement of refugees in particular and, less so, of skilled migrants. Such local government initiatives range from Multi- and Intercultural policies and initiatives such as Intercultural Ambassador Programs^4^ to involvement in larger scale initiatives such as the EU Council-initiative “Intercultural Cities”^5^ or Australia’s own incarnation of the “Welcoming cities”-initiative (Wickes et al., 2020). The aims of these initiatives include the social and cultural integration of “culturally and linguistically diverse” arrivals^6^; enhanced intercultural engagement between “existing” and “new” residents; addressing structural barriers and insufficient ethno-cultural resources for new arrivals (Jordan et al., 2007); and improving social cohesion in increasingly culturally diverse communities (Moran and Mallman, 2015). A rationale underpinning many of these initiatives is to increase the retention of migrants in the location (Krivokapic-Skoko and Collins, 2016), thus focusing on those migrants who have a legal pathway to a longer-term residency.

The growth of intercultural and multicultural policy investment in regional Australia has occurred largely in parallel to the earlier mentioned de facto labor migration programs such as the “backpacker”- visas^7^ but also the dedicated seasonal worker program, which are shaped by the seasonal demands for agricultural labor. Notwithstanding the different aims underpinning regional refugee settlement (economic) and regional multi/interculturalism initiatives (cultural, social) on the one hand and visas for transient migrant labor (economic) on the other hand, these policies have jointly led to the co-existence of a wide range of residents with migrant backgrounds in regional locations and labor markets. While there are migrant and refugee groups whose retention in the regional location is an objective of local governments, there are also transient migrant workers whose readily available labor is critical to local businesses but whose existence is ignored in local multi- or interculturalism policies. Despite the absence of this latter group from most local governments’ concerns with social cohesion and local multi- or interculturalism, transient migrant workers contribute significantly to local regional and rural development both through their labor and their consumption in regional towns. Their economic significance has been evident in the exceptions made for seasonal workers whose visas were extended in the context of Covid-19 (Sullivan, 2020), while PM Morrison told other temporary migrants to “go home” if they could no longer support themselves in the face of lost work due to business closures (Gibson and Moran, 2020).

Revisiting how the relationship between migration and development is conceived in the different policies described here, the economic rationale of Australia’s overall migration policies (Wright, 2015) clearly shines through. The development objectives underpinning the skills and labour-focused regional migration policies at the federal level (i.e., the skilled temporary and regional skilled visas, the WHVs, and the Seasonal Worker program) are primarily economic via increasing a flexible labor supply and specialist skills basis in regional Australian towns. Regional employers and businesses and local economies clearly benefit from regional migration. This purely economic reading of migration benefits mirrors the conventional understanding of “development” in the (international) migration-development nexus, which is “firmly rooted in understandings of development as economic growth, which privileges the productive dimension of migrants’ lives” (Bastia, 2018, 471). In the context of international development, the economic development is expected to accrue to migrants’ countries of origin through investment of remittances. This economistic focus in development has received much criticism with scholars pointing to the many social and cultural development outcomes migration can achieve as well as the call for a focus on migrants’ development (Piper, 2009). In the mentioned regional multiculturalism and interculturalism-focused initiatives at the local and partly, the State-level the focus is, on the other hand, on the social and cultural outcomes of regional migration, with the beneficiaries being ideally both, the newly arriving migrants and the regional communities, in which they settle.

## Scholarship on Migrants’ and Refugees’ Regional Migration Experiences in Australia and Internationally

Australian scholarship on regional migration has grown since the early 2000s, alongside international research on rural migration in the global North. Starting with early analyses of the potential for and trend of regional migration (Withers and Powell, 2003; Hugo, 2014b), government-commissioned evaluations of specific regional refugee settlement pilots (Piper and Associates, 2007; Shepley, 2007; Piper and Associates, 2009) and studies of early refugee relocation initiatives (Taylor and Stanovic, 2005; ICEPA, 2007), the questions of attraction and retention, and “what works and doesn’t work” in regional migration and particularly in refugee settlement has inspired much research.

Research on the experiences of migrants and former refugees in regional Australia and elsewhere has also been shaped by different disciplinary and epistemological priorities and foci. Much scholarship falls into one of two categories: studies that have focused on the employment experiences of migrant workers and, less so, of former refugees; and studies that are primarily interested in social and cultural dimensions and impacts of rural migration and settlement such as intercultural relations, social and cultural integration, belonging and in- or exclusion of migrant arrivals. Rural sociologists and human geographers have perhaps most successfully combined analysis of economic, social and cultural impacts of migration and mobility, identifying the emergence of an increasingly multicultural and multifunctional “global countryside” (Woods, 2007; Argent and Tonts, 2015). Each body of scholarship has contributed important insights that need to be brought to an analysis of the relationship between regional migration and refugee settlement and development.

Agricultural and horticultural jobs and employment in related food production sectors are the most-researched areas of regional and rural employment of migrants in Australia and internationally. Many researchers across the global North have highlighted the exploitative employment conditions migrant workers are habitually submitted to on farms, in orchards and in food processing, enabled by regulators’ neglect in securing labor standards and a blind eye to unlawful employer practices in the context of a restructuring agricultural industry (Rye and Scott, 2018; Rosewarne, 2019). Wage theft, unsafe and unhealthy working conditions, and physical (including sexual) abuse of migrant workers in workplaces that are generally “out of sight” and often neglected by law enforcement have been identified time and again, in studies in the United States (f. ex. Holmes, 2013), in countries across Europe (Rye and Scott, 2018), the United Kingdom (f. ex. Rogaly, 2008) and also in Australia (see f. ex. Underhill and Rimmer, 2016; Howe et al., 2020; Reilly et al., 2018). (Socio-) legal scholars and sociologists have drawn attention to the detrimental role of temporary migration regulations and citizenship status in producing or exacerbating precariousness in employment for many migrant workers (for Australia, see Howe et al., 2020; for Canada, see Preibisch, 2007; for Italy, see Urzi and Williams, 2017). Development scholars and political economists have furthermore queried the development impacts of guestworker schemes in the agricultural sector such as Bracero schemes in the United States and Canada (Basok, 2000; Preibisch, 2007) or the seasonal worker program in Australia (Rosewarne, 2019). In addition to this important research, relatively fewer studies have attended to the employment experiences of regional migrants in “skilled visa”-categories and of former refugees. An important finding here in Australian research has been the lack of employment pathways in regional locations, which has affected both groups (Johnston et al., 2009; Boese, 2013; Schech, 2014; Curry et al., 2018), whether based on racial discrimination or a scarcity of higher-skilled job openings.

The social and cultural impacts of regional migration, and the regional settlement experiences of migrants and former refugees have been another key focal point of research on rural migration over the last 2 decades. Research in Australia reflects trends in other locations. Overlapping interests have been in the transformations of rural places through migration, which have been captured through various sociological concepts including social integration, belonging (De Lima, 2012), and social cohesion (in Australia, Moran and Mallman, 2019), rural multiculturalism (Wilding and Nunn, 2018) and rural cosmopolitanism (Schech, 2014; Woods, 2018a) (for which Woods (2018b) has identified early beginnings in late 19th and early 20th century Australia). Scholars in Europe and Australia have highlighted the interconnectedness of rural places with others through the concept “translocalism” (De Lima, 2012) and “multi-local settlement” (Boese et al. 2020). Building on early research on rural racism (Chakraborti and Garland, 2004), researchers have explored the experiences of minority ethnic and racialized groups in rural locations through notions of visibility (Galligan et al., 2014), “intercultural encounters” and “everyday otherness” (Radford, 2016, Radford, 2017), and, importantly in Australia, the embedding of “local hierarchies of racialised and classed belonging and exclusion” in settler colonialism (Butler and Ben, 2020).

A growing body of research has highlighted limitations of current regional migration policies in Australia, pertaining to each of the mentioned groups as defined by visa status–refugees, skilled migrants, seasonal workers and backpackers. Two key insights that emerge repeatedly from these critical analyses are the challenge of limited employment opportunities for skilled migrants and refugees (for example; Johnston et al., 2009; Schech, 2014; Curry et al., 2018) and the persistent exploitation of those who find work, in particular those on temporary visas and those working in the horticultural sector (for example Underhill and Rimmer, 2016; Reilly et al., 2018; Howe et al., 2020). Recent scholarship has also stressed the historical embedding of labor market-based inequalities and exploitation (Butler and Ben, 2020). These analyses highlight the need to unpack the nexus between migration and regional development further.

To do so, we suggest considering the question development scholar Raghuram (2009) has raised to remind international development practitioners and scholars of an understanding of migration “as personally developmental, rather than nationally developmental”: “which migration? What development?” (Raghuram, 2009, 103). While Raghuram aimed to draw attention to the personal losses migrants might face who focus on development “there”, referring to their home countries, we suggest considering the losses migrants might face in the course of regional development “here”*,* in Australia.

## Research and Methods

The data discussed in this paper stems from three interview and focus group-based research projects conducted over the last 10 years by one or both of the authors in rural and regional Victoria, in Australia’s South East. These projects cover a diverse range of regional and rural locations in Victoria, in terms of their town size, migration patterns and history, and economic profile. Each project focused on regional migration, its impact on regional towns and on regional migrants themselves. We will foreground findings that raise fundamental issues and questions when researching the nexus between regional migration and development outcomes for migrants in a society of the global north that is built on migration and has seen the development of multicultural policies at all levels of government (Federal, State/Territory, Local). We will highlight evidence that directly addresses this paper’s key research question “What kind of development can regional migration afford to migrants?” And, we will reflect on how regional migration policies impact migration experiences.

The first of these studies focused on regional settlement of recently arrived migrants and refugees to rural Victoria (referred to as Regional Settlement-project). It was one of the first research projects in Australia to investigate this phenomenon through a study of six regional and two metropolitan locations with data collected between 2010 and 2012. The project pursued a twofold focus on the intergovernmental coordination of regional migration and the settlement experiences of recently arrived visible migrants and refugees, including analysis of their employment pathways and identities. It was guided by the question: What are the social, economic and political factors that affect the resettlement experiences of recent visible migrants and refugees? The research comprised of an online survey of 106 settlement stakeholders, in-depth interviews with 85 recently arrived migrants and refugees, key informant interviews with 47 stakeholders and 14 focus groups with a total of 90 stakeholders involved in regional settlement, from government, business and the community sector. This included members of local settlement planning committees such as multicultural liaison officers in local government, the health sector and the police; key employers and service providers. The data we draw on in this paper is from the interviews with migrants and the focus groups and interviews with key informants. The research locations were selected to reflect the diversity of settlement locations in terms of their size (large regional towns and small rural locations); immigration histories (“new” vs. “old” settlement locations); and the composition of recent arrivals in terms of migration streams (skilled, family, humanitarian); and regions of departure (Middle East; Asia and South-East Asia; Africa).

The second, unrelated project (referred to as Regional Multiculturalism-project, data collected in 2015) focused on multiculturalism and social cohesion in two rural communities (populations approximately 30,000 each) conducted in collaboration with a government agency. At the last census in 2016, each town had over 10% of its population born overseas. The towns’ horticulture and agricultural industries have attracted waves of migration since the late nineteenth century and have recently relied heavily on temporary migrants for harvesting, picking and packing. Each location has also experienced relatively large influxes of humanitarian visa and asylum seeker migrants since the 1990s. The project examined factors enhancing and militating against social cohesion in multicultural contexts, guided by four main research questions: What are individuals, community-based organizations, local government, policy makers, and businesses doing well in terms of “getting along” in a multicultural/ethnically diverse environment? Where and what are the problems that affect social cohesion? Are there groups of people in particular difficulty? Are there current, emerging or foreseeable tensions between different people? And what might be done about them? This project involved 78 formal in-depth interviews, four focus groups, and also observation and informal conversations at community events such as festivals and film nights, and at community meetings. Researchers spoke to people from a range of health, welfare, government and non-government agencies, as well as community members from a range of immigrant, non-immigrant and Indigenous Australian backgrounds. Given the ethnic diversity and central importance of migration history in these locations, issues of migration loomed large in discussions about multiculturalism and social cohesion.

The third project (referred to as Regional Mobilities-Project) was developed on the basis of the authors’ respective preceding research and was conducted jointly in 2016–2017, It focused on the intersection between social and spatial mobilities of migrant workers in rural Victoria, taking a town with a population of approximately 30,000 as case study. The research questions were: How do migrants’ social and spatial mobilities in regional Australia relate to each other? How is social mobility shaped by spatial mobility, and vice versa? The study included 18 in-depth interviews with people from international migrant backgrounds, and 10 in-depth interviews with key stakeholders with knowledge of and experience with migrant settlement issues, all living in the main town and nearby small towns. The international migrant participants were skilled migrants, humanitarian/refugee/asylum seeker entrants, and WHV-holders. Key stakeholders were from the community sector, local government, education sector and a large business. Interviews with international migrants focused on geographical moves and employment pathways within Australia, eliciting perceptions of motivation, interpretations of outcomes, and articulations of hopes and aspirations, for work, family, social mobility and possible future moves. Experiences of belonging and exclusion were also discussed. Key stakeholders were asked for their perspectives on migrants’ geographical movements and experiences of employment, inclusion and exclusion, barriers and opportunities.

## Unpacking the Nexus Between Regional Migration and Development

Our research on regional migration experiences reveals the complex relationship between policies, social and economic hopes, needs and aspirations. Migrants on different visa categories and from different socio-economic and cultural backgrounds move to regional locations with varying intentions and hopes, structured by and structuring experiences of time and place, of regional settlement and mobility, and indeed of “settlement mobilities” (Boese et al., 2020). In turn, the development outcomes of their migration can also be assessed along the axes of time and mobility. Within the wide range of regional migration and settlement experiences captured in our research, we can identify three sets of findings that illuminate the complexity of the regional migration-development nexus.

### Employers’ Role in the Regional Development-Migration Nexus

Our first set of findings addresses the significant and complex influence of employers on the development outcomes of regional migration, applying our focus on migrants’ development. . Firstly, employers’ demand for workers shapes migrant flows to regional locations and migrants’ experiences in those locations in manifold ways. This is true for nearly every category of migrant, from skilled visa holders to backpackers and permanent residents from refugee backgrounds. For skilled workers, employer demand serves as the entry ticket to a regional skilled visa. Vacancies on farms and in regionally based cafes and restaurants meet a steady flow of WHV-and Work and Holiday visa holders (albeit disrupted by the arrival of covid-19). Seasonal workers from the Pacific Islands move to where employers participating in the Seasonal Labor Scheme need them. Finally, many former refugees move to regional Australia because of relocation initiatives including specific regional employers.

Secondly, employers directly shape migrants’ experiences in regional locations beyond the point of attraction, thus having an impact on migrants’ development outcomes. As sponsors of the majority of skilled-visa holders and also as employers of former refugees who relocate to regional towns, some employers take on important pastoral roles. The findings from the Regional Settlement-project highlighted that migrants not entitled to settlement support often viewed their employer as the first port-of-call in any situations requiring local knowledge and advice. A group of former international students from India who had since been sponsored for a regional visa, characterized their employer affectionately as “google.com”, signaling his role as go-to-person for any arising problems, from purchasing a car to finding suitable accommodation:

*Sameer:* And every week, if we have some problem or we need something we go to Bruce …

*Amrit:* If anything bad happened we call Bruce, anything could happen, we call him.

*Balraj:* Car breaks down, he comes and picks it up.

Similarly, some of the former refugees who relocated from Melbourne to regional Victoria to take up employment in a meat processing plant identified their employer as their first point of contact when any problems arose. In one case this included helping out when a conflict with police arose due to a driving offense. In another case the employer’s support consisted in speaking up publicly against the racial vilification one of his employees was subjected to outside of his workplace.

The critical role of employers in the settlement and welfare of migrant workers was also highlighted by other stakeholders in the often tightly-knit regional communities:

The one thing I guess that stands out in the community for us, is that in a rural setting, a regional setting, the role of the employer is significant in the settlement of people of refugee backgrounds or migrants. Whereas in a big city like Melbourne … the role of the employer is different. There are so many other supports and other … opportunities and varied experiences, whereas in a place like (name of town) the role of the employer is significant.

Some employers themselves similarly characterized their relationship to migrant workers who they had attracted to the regional town in ways that indicated a paternal sense of responsibility for their settlement and a more personal relationship. One employer who also appreciated learning about different cultures illustrated this:

I sort of felt that if I had kids that were travelling around the world, you would hope that there would be somebody who would take an interest, help them if they got into a little bit of trouble.... So I end up becoming their substitute parent. I mean, the Indians here, they call me uncle. So whenever there is a problem they ring me up “can you help move some furniture, what do we do with this … ”

In the Regional Multiculturalism-project, a local employer, who had successfully expanded his family-owned business by embracing migrant workers in a context of labor shortage, also reflected on a sense of obligation to do something to help integrate the new migrants who had moved into his area:

I ended up with African neighbors over the road. My kids went to school with African kids. It was a big and sudden change in the community that opened my eyes, and at the same time I was going to work faced with 10 applicants none of which were suitable or ready for work. So we literally went through a process of sitting down with our whole staff in a staff meeting and saying, “We’re going to do something different where we’ve got these new people in our community and we want to give them a chance. We want you to embrace them. We want you to make the effort to extend and help these people settle in our workplace.”

This employer was well known in the area for employing a high number of migrant and refugee employees, on decent wages and conditions, and with strong anti-discrimination and anti-racism work practices.

It was clear from the accounts of several migrants, some employers themselves, and other key stakeholders, that the very act of having “brought” migrants to the location often came with a sense of responsibility. In particular in smaller towns with predominantly white Anglo-Australian local populations for whom the sudden influx of visibly different migrants was a first time experience, local governments and some local service providers were left with a sense of being overwhelmed and unprepared. In one of these locations the employer was a regular participant in the local settlement planning committee because of his intermediary role in relation to recent arrivals; in other locations, employers became vocal advocates for attracting migrants and refugees to the location, based on their experience with an increasingly culturally diverse workforce.

Employer practices varied a lot, however, and some employers’ sense of responsibility should not be confused with, and does not replace, accountability. On the contrary, the lack of accountability emerged in examples where migrants described being underpaidor discriminated against, which most tolerated without complaining (as is often observed in studies of precarious migrant labor (see f. ex. Rogaly, 2008). The combination of quasi-settlement support and exploitation in the same employer both challenges a neat qualification of employment as rural settlement “success” (Curry et al., 2018), and raises questions about the relationship between exploitation in employment and rural conviviality (Neal and Walters, 2008). The constellation of employers who step in as quasi-settlement support workers while benefitting from workers’ compliance, also differ from the kinds of bonds between employers and migrant workers described elsewhere (Rye and Scott, 2018). They demonstrate one of several side-effects of an employer-oriented migration policy that prioritizes local economic interests (or capital) over migrants’ welfare. The fact that employers play such an important integrative role also reveals, as argued earlier, how categories of migrants are constructed through government policy regimes as labor resources, for regional areas, whose own professional development needs are secondary to the imperative of economic development.

Thirdly, when considering employers’ shaping of the regional migration-development nexus, not only do they directly affect migrants’ socio-economic situation through levels of pay, units of labor (piece or hourly rates) and hours, they also shape workers’ employment satisfaction, their work-based identities and future employment pathways. How much significance migrants themselves attach to the latter depends on several factors, of which two stand out as particularly relevant for the broader question of development. One is the meaning of a specific job in relation to migrants’ employment trajectories and overall life courses; the other is the overall purpose of their migration to Australia. The next section will explore how these two interrelate.

### Regional Migration in the Context of Migrants’ Life Courses

To assess the development outcomes of regional migration with a focus on migrants themselves requires a consideration of how migratory movements are embedded in migrants’ life courses. Migrants move to regional Australian towns for different reasons and with varying expectations, needs and aspirations. WHV-holders, for example, primarily seek to find short-term work on farms that is relatively easy to access, not requiring prior experience nor an arduous application process, and is often combined with the provision of accommodation. As explained earlier, a second-year on the WHV can be accessed by working in a designated regional location for 88 days. Backpackers’ stays in regional Australia are thus relatively short-lived, even if it commonly takes many more than 88 days of residing in regional locations to accumulate 88 days of work, due to weather conditions and the insecurity of shifts. The experiences of backpackers in regional Australia tend to involve continued mobility in search of the next job, expectations tend to be focused on work, as this quote from an interview with a British backpacker, from Regional Mobilities-project, illustrates.

I’m aiming to get to Byron for June and July. Because that’s when the blueberries start again. I think in blueberries you can make the most money picking fruit, cause it’s so small, it doesn’t weigh a lot, if you do it quite fast. But I’ve got till June, July, so hopefully I’ll find a farm where I can do it, just so I can tick it off my visa.

Even if pay rates are poor, and backpackers are often subject to unlawful labor conditions including wage theft and even physical and sexual abuse as has been widely reported in the media and scholarship (see f. ex. SBS, 2015; Farbenblum and Berg, 2017; Mullins, 2019) many backpackers stay on to accumulate their 88 work days to secure the entitlement for a second year visa. Here we see a direct relationship between policy regime (requirements to attain extension of visa), regulatory neglect (lack of government action to improve labor conditions for backpackers) and outcomes for this migrant category. Overall, the working holiday-period constitutes an extra-ordinary experience in the lives of many backpackers, *not* related to their lives in their home countries, neither in terms of building on their previous experience or qualifications, nor in terms of working toward their employment future. The regionally based manual laboring job is for many a mere “stint” in their lives, that some interpret and validate retrospectively as resilience-building experience, and at best a quick source of income. One British backpacker in the Regional Mobilities-project explained his reasoning for the initial move to Australia as follows:

I think I was quite fed-up at home. I felt like I was stuck in a rut. Also seemed to kept being dragged into other people’s dramas. I suppose I just wanted to get away from (it all) to be honest with you, and just have a break. At the same time, you just want to go to Australia. (laughs)

Backpackers commonly travel with others and find out about the next farm job from co-workers, co-travelers or via social media. They often stay in “backpacker hostels” that are let to them by labor contractors or on the farm, and barely engage with local communities. Their contribution to Australia’s horticultural production and to local labor supply is significant, while their social and cultural impact beyond the economic dimensions of regional development is minimal. This reflects research findings on rural migrant workers in food production in other countries (Riley et al., 2018). Mostly out of sight of the local community, backpackers are perceived as an anonymous yet considerably sized transient population that is inconsequential for most locals, although some stakeholders cautiously indicated that the flux of backpackers is not always embraced by the local population. As one stakeholder in the Regional Multiculturalism-project commented, “They’re a little bit of a disruptive influence.” It is crucial to note here however, that the availability of a perpetual flow of mobile labor through backpackers (at least until the arrival of the Covid-19 pandemic), means that in some regional locations refugee settlers who seek local work compete with this more mobile labor supply. So the regulatory production–via the WHV–of flexible, willing and readily available flows of workers who are extremely unlikely to seek enforcement of their labor rights, harms the regionally resettled refugees who try to access low-threshold jobs on farms, and benefits the farmers or contractors who can employ at the cheapest possible rate.

Many regional residents with migration or refugee backgrounds move to regional Australia with the aim of establishing a new home. This kind of migration for settlement is usually connected to the hope for, or the specific prospect of, paid work but the migration decision is often also infused with expectations of a particular lifestyle, a desire for a sense of belonging and overall the hope for a better life. Across all three research projects we met people from refugee backgrounds who moved to a regional area to satisfy their desire for a safe space for their young children to grow up in, away from the risky influences they associated with major cities, as this typical statement from a former asylum seeker in the Regional Mobilities-project indicates:

If the children from age 16 to 21, they are free to go outside day and night to contact with bad people, for example, who are drunk or addicts or trouble maker, certainly they will get in trouble for that finally. The environment is very important for the family (in this town), for the children.

Another important dimension of migration that relates to hopes, needs and aspirations is the variety of experiences within one household. All of our research projects included spouses who compromised their career through the move to a small regional town. Highly skilled and qualified but without an employer sponsor, many partners of temporary skilled visa holders cannot find suitable employment and prioritize their partner’s employment and their children’s wellbeing over their own career. This example not only indicates the more limited labor market opportunities in small regional towns, but it also highlights the importance of studying migration policy outcomes beyond the level of the primary visa holder, and of considering the significance of family and household needs in shaping retention. There are costs attached to these compromises, which rarely show up in the “win-win” accounts of regional migration. This is evident in accounts like the following from a highly educated spouse of a skilled visa-holder from India who participated in the Regional Settlement-project:

The fact is when I was walking from my home to (the supermarket) for my very first day, the very first day when I had to start my job, I was crying … I had tears in my eyes, and I was thinking my qualifications … I’ve completed my double masters from India, I’ve done double bachelors and double masters, and I was doing another masters, I’m still doing it, from University of Southern Queensland, and that’s Masters in Education, and on top of that I have nine years of teaching experience and all in good schools which were reputed to international standards. And I was thinking that I worked so hard all my life, and I worked in the top-most industries and in the top-most school, never ever thinking that one day I’ll stand here serving people at … the front desk.

Beyond the despair and emotional burden of underemployment, the considerations of another highly qualified participant, a young woman from South East Asia and participant in the Regional Mobilities-project, demonstrate the importance of medium-term aspirations beyond life in the regional town alongside the short-term prioritization of the children’s needs for stability.

I mean, we didn’t want to uproot, move kids again, we’ve made that big move from overseas to here, that’s a bit too much. That also is the reason why we thought maybe we’d settle in (this town) in the meantime, because we don’t want to uproot her again. To be honest, we’re thinking three years’ time she’ll be going to Uni, and then maybe we can make another decision. So we could maybe move.

Others again move for the job their visa is tied to, and they expect to move again, maybe three years later, when their legal status provides them this freedom. Or they have a clear earning objective in mind, and they will move on again once they have saved sufficient money to achieve their real objective, as illustrated in the following account of a recently arrived refugee (in the Regional Settlement-project) who has picked up work in a meat processing plant to earn money for his visit home:

To me it is a temporary work. Because I have a reason why I have come here. The reason is simple as I just want to pay for my ticket and leave. The temporary work for is actually, because I still have the dream of going back to university and finishing my degree.

Across the different groups it is clear that regional migration and its meaning in people’s lives vary considerably depending on the temporal embedding of regional migration in people’s life course and the hopes invested in this migration. How paid work features in the overall experience of migration is a lot more variable than a purely economic interpretation of the migration-development nexus would suggest.

### Long-Term Pathways for Sustainable Development Versus Short-Term Fixes

A third set of findings relates to the tension between short term fixes–primarily of labor or skills gaps–and long term planning for sustainable settlement including pathways for regional migrants. Short-term regional migration planning that starts and ends with matching willing workers with vacant jobs often comes along with underemployment, if not for the primary visa holder, then for their partner. Underemployment is a well-researched and widely known aspect of recently arrived migrants’ and refugees’ employment trajectories (Ho and Alcorso, 2004; Colic-Peisker and Tilbury, 2006). Taxi-driving engineers have become a cliché illustration of the wide-spread occupational down-grading or “skidding” (Hugo, 2014a) and status loss experienced by many tertiary-educated new arrivals, in particular from refugee backgrounds, in Australia (Constable et al., 2004). In addition to the mental health impacts such downgrading can have (Das-Munshi et al., 2012), the issue of blocked employment pathways also obstructs sustainable regional development. Alongside the already mentioned temporary compromises or trade-offs migrants might engage in at the level of employment, migrants also vote with their feet and leave regional locations that do not offer career pathways.

Service providers who are at the coalface of assisting migrant jobseekers to enter the labor market are often well aware of both the obstacles many of them face and their aspirations and dreams. Many employment service providers today embrace the workfare mantra that “any job is better than no job” or even advise their clients to take on voluntary work in order to improve their opportunities for paid work in the long run. The Australian Government’s Cultural Orientation Program (AUSCO) advises refugees even before they arrive in Australia that “learning English and accepting the first job you are offered are the first steps (in) settling into your new home” (DSS, 2018: 1). Several of our research participants across the three projects have heeded the advice to volunteer or decided themselves to choose that path when failing to find a paid job.

Pascal, a former refugee from Togo and participant in the Regional Settlement-project, explained that a settlement service provider sometimes drew without paying on his interpreting support when they conducted research. Susan, a former refugee from Ethiopia who participated in the Regional Settlement-project, volunteered in a childcare center. Lilly, a skilled migrant from South East Asia and participant in the Regional Mobilities-project, quoted below, started volunteering in a local community organization, “to get to know people”:

We felt that for us to fast track integrating into the community, we need to really get out there. I mean we knew of (our own) community around the area, but we felt like if we just stuck to them, I mean, we can’t move on. We need to know the culture, we need to know the language, we need to know the people and understand how the community operates.

The leader of a local Congolese association, from a refugee background and participant in the Regional Multiculturalism-project, who had worked in high status professional jobs in Africa, spoke of his typical combination of volunteering and part-time work: “(W)*i*th all my work I’m doing here, … I never work as a full time...I always work as volunteer, I work I guess part time so, but I’m casual.” He reflected that this was a common experience among his migrant and refugee peers.

Several women we interviewed for our research on the relationship between social and spatial mobilities explained that their volunteering work contributed to their sense of belonging and connectedness, and in some cases even to paid work. It is clear from the way in which volunteering is represented by service providers but also by many volunteers themselves, that many perceive it instrumentally as a vehicle of development at the level of the individual migrant. This includes the development of social networks, familiarity with local people and workplace culture and a sense of belonging. While lacking financial returns or indeed a guaranteed pathway to paid work, the sense of contributing and gaining connection makes volunteering worthwhile for many regional residents with migration or refugee backgrounds, especially when they have a partner in paid work.

Others can however not afford unpaid work and have to take on any paid job they can access. In regional locations, these typically encompass a range of hard, manual labor, from farms to packing sheds or at the local meat plant, the kind of employment known as precarious, low-paid, insecure and often unhealthy. Depending on migrants’ aspirations and their migration or relocation intentions, such jobs may be calculated to achieve a necessary income or interpreted as a temporary solution until a better job comes up. At the same time, shift work often interferes with other professional development activities, such as the participation in an English language course, thus obstructing the pathway to a better job, as is illustrated by Wei from China (participant in the Regional Settlement-project), a skilled migrant who works at the meatworks: “the problem for most new migrants, as well as my personal circumstances, is I would like to improve my English, however in the mean time I have to work so there’s a conflict.”

The dead-end nature of many jobs in regional Australia, that are increasingly filled by migrants or former refugees, is no secret to regional stakeholders who see people leave because of the lack of employment pathways. Already in 2011, a peak service provider in regional Australia who took part in a focus group with other key stakeholders in the Regional Settlement-project, drew attention to the potential detrimental effect of lacking pathway planning:

And it’s a pathways question too cause … the sad reality is that a lot of people will have to start their life in Australia in a meat factory … or driving cabs but, but that if, if, we are to avoid the situation where you do get an underclass in rural Victoria I think we really need to think about the pathways out of those places and I, and I think to its credit although it may not be the case at the moment I think (an earlier relocation initiative) may not have succeeded but at least it had the aspiration to, to create those pathways when it, when it first started.

The notion of a rural social underclass has also been raised in studies of migrant workers in rural food production in other countries (Rye, 2014), highlighting likely limitations of rural migration outcomes.

The Congolese association leader (interviewed for the Regional Multiculturalism-project), quoted previously, also explicitly emphasized the way that the local Anglo-dominated community where he lived, and its local council, were prepared to celebrate cultural diversity, but that employment-related questions were thoroughly neglected:

And in that context, you can see that the social inclusion in (this town is) becoming hard. (This town) only does good … when there is a function, when you invite people there is a party you know? So you can see people came from, because where everyone had come and they want to demonstrate this (culture) and dance and everything, but when it came to employment, that is where it becomes issues.

This town is frequently cited in the media as a successful multicultural place, but when asked about this, he viewed the town as unsuccessful, because it did not provide economic opportunities for people from many migrant and refugee backgrounds. While local council had a cultural diversity strategic plan, it had no strategic plan for employment. In addition, there was no local interest in drawing on the experiences and knowledge of refugees:

So the question is you have, or we use, because this is the idea of settlement, if you look at how’s the settlement started, it was to bring people so we can share knowledge. So why this has been stopping for the recently immigrant? We don’t see if there’s much effort that’s put in place to help immigrant to use all kind of knowledge they brought from overseas, here. They want to contribute in this region as well.

Another Congolese former refugee from the same town (participant in the Regional Multiculturalism-project), who had extensive IT experience in Africa, could only ever get short term work, and did a lot of volunteer work at schools, even though he had added an Australian Masters in IT to his previous skills. He spoke of his continual struggle to find paid work, despite his high level of professional skills; he felt that employers would use the three or six month probation period to enact a hidden form of discrimination–though he had good reports on his work, he had had more than one experience of his contract not being renewed after the probation period had finished: “I believe that when they don’t take you for the full time or they don’t give you another contract and they don’t tell you which kind of mistake you did so that they cannot keep you, there is hidden discrimination”. Though his wife and children were settled in the town, and his wife preferred to stay there, he was thinking about moving to a bigger city for opportunities.

## Conclusion

Australian governments since the 1990s have made concerted efforts to attract and retain a range of migrants and refugees in regional Australia, aimed at economic development and sustainable populations for rural communities, while at the same time taking pressure off larger cities and their infrastructures and services. They have introduced a range of visas among other policy initiatives to encourage regional migration including of highly qualified “skilled migrants,” “flexible, mobile labor” for regionally based unskilled jobs and resettled refugees. Our analysis has demonstrated that the majority of these policies treats regional migrants as agents of local economic development via their participation in regional labor markets, which is reflected in the persistent, wide-spread exploitation of transient horticultural workers, the insufficient consideration of settlement support needs for non-transient workers; and the lack of concern with employment opportunities for refugees and partners of skilled migrants. While these different groups have received separate attention in studies of rural migration, their experiences have rarely been analyzed together, and with a focus on their own development. Thus, studies of exploitation of migrant and refugee workers in regional industry sectors (as discussed in many destination countries in the global North, see Rogaly, 2008; Underhill and Rimmer, 2016; Rye and Scott, 2018) implicitly challenge the notion that regional migration benefits migrants, but such findings are rarely used to challenge the positive development-outcomes of regional migration, promoted by policy makers, researchers and settlement service providers alike (AMES Australia and Deloitte Access Economics, 2015; Collins et al., 2016; Tudge 2019). Blocked employment mobilities whether for skilled migrants (Schech, 2014) or for refugees (Curry et al., 2018) have slowly become recognized as skills wastage and wastage of human capital (Constable et al., 2004), but the reference point for these losses is often local economies rather than people with migrant or refugee backgrounds themselves. We argue for a shift in measuring the development outcomes of regional migration to centering migrants’ development as a benchmark for successful regional migration policies.

Our analysis of interviews and focus groups conducted over the course of ten years has identified three important dimensions of regional migration that illuminate the limitations to viewing regional migration as a source of development: the complex role of employers; the embedding of migration events in migrants’ life courses; and the tension between short fixes and long-term pathways. The influence of employers beyond the initial attraction and employment of migrants is significant for understanding their stake in regional migration and retention and, consequently, in the regional migration-development-nexus. Despite employers’ role in attracting and often retaining workers, and their variable role in settlement support, employers are hardly guarantors of migrants’ and refugees’ development in regional locations. Some employers have stepped in to fill the vacuum created by governments, characterized by lack of planning and the inadequacy or absence of local, targeted support services, that also contributes to the absence of viable employment pathways for economically, socially, culturally and racially vulnerable migrants and refugees. This situation in combination with the extensive exploitation of the labor of migrants and refugees, especially in horticultural work and food processing, also threatens regional development. Not only does the underemployment of many migrants and refugees experienced in regional Australia translate to a loss of skills from the perspective of local economies but the lack of avenues for skills development and the absence of viable career pathways, ultimately affect the retention of migrants.

The policy failure of, on the one hand, viewing skilled migrants as simply “needed” for their skills while neglecting to support their and their partners’ integration and socio-cultural inclusion, and on the other of viewing refugees as those who will and should accept the first job on offer (DSS, 2018), and as primarily requiring a policy response of cultural “integration” because they are seen as “cultural Others”, also has negative implications for regional development. It has effects on the development of migrants and refugees themselves (Piper, 2009), resulting in thwarted ambitions and hopes for many partners of skilled migrants and blocked pathways for many refugees, exacerbated by the poor employment conditions that characterize work in typical regionally based employment sectors such as horticulture and meat processing. The implications of WHV failures in combination with poor labor law protection in the horticultural sector have been highlighted elsewhere and combined with calls for visa reform and better rights protection (see f. ex. Farbenblum and Berg, 2017; Reilly et al., 2018, Campbell 2019), but the effects of these policies for other locally settling migrants and former refugees need to be much better understood. The discussed findings from three studies of regional migration conducted over the last 10 years, indicate that the question, what development regional migration affords to migrants themselves, deserves more attention in future regional migration policy design.

## Data Availability

All relevant data is contained within the article: Selected original contributions are included in the article. Raw data cannot be made available in its entirety based on the limitations of the university’s research ethics approval and participants’ consent. Further inquiries should be directed to MB, m.boese@latrobe.edu.au. Requests to access the datasets should be directed to MB, m.boese@latrobe.edu.au.

## Footnotes

1 In this paper “regional migration” refers to both direct migration from overseas and secondary migration of people with migrant backgrounds from another place in Australia to a rural or regional location. The latter category includes people from forced migration backgrounds, and in terms of their legal status this means recognized refugees, humanitarian visa holders and asylum seekers. For the purpose of regional visas, the Australian Department of Home Affairs classes “most locations of Australia outside major cities (Sydney, Melbourne and Brisbane)” as designated regional areas for migration purposes.

2 The term “migration-development nexus” describes the relationship between migration and development, in particular the impact of migration on development, which has been debated for several decades and variably interpreted by policy makers, practitioners and scholars (see f. ex. Bastia, 2018).

3 This program is designed to allows temporary circular migration of nationals from nine Pacific Island countries and Timor-Leste to work for employers in the agriculture and accommodation sectors.

4 See f. ex. https://www.bendigo.vic.gov.au/Services/Community-and-Care/Intercultural-Ambassador-Program

5 See f. ex. https://www.ballarat.vic.gov.au/city/my-community/intercultural-city

6 This phrasing and the acronym CALD are commonly used in Australian policy and practitioner discourse.

7 “Backpackers” is the common label for young people on Working Holiday- and Work and Holiday-visas in Australia.
